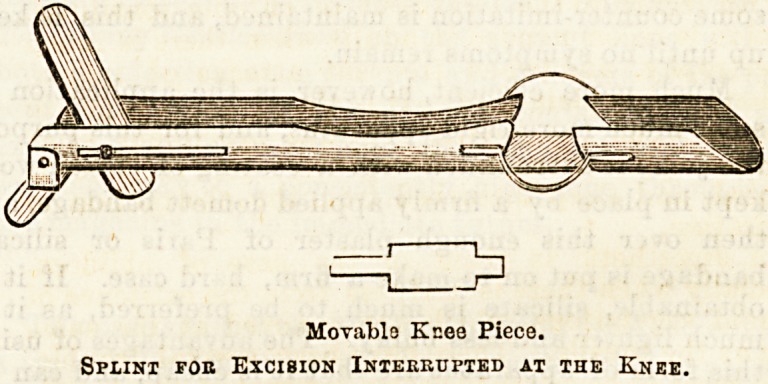# The Treatment of Strumous Disease of the Knee-Joint

**Published:** 1893-01-14

**Authors:** 


### THE HOSPITAL CLINIC.

[*The Editor will be glad to receive offers of co-operation and contributions from members of the profession. All letters should be addressed to* THE EDITOR, THE LODGE, PORCHESTER SQUARE, LONDON, W.]

### ST. BARTHOLOMEW'S HOSPITAL.

### TREATMENT OF STRUMOUS DISEASE OF THE KNEE-JOINT.

In dealing with the treatment of strumous or tubercular knee-joint, we shall only speak of the methods most commonly adopted at St. Bartholomew's Hospital. These methods may be conveniently described under two headings—(1) Local; (2) Constitutional.

1. *Local Treatment.*—Under this head, the cases may be divided according to whether they are treated without or with operation.

First, as to those treated by expectant methods. The essential here is to maintain, as far as practicable, perfect rest to the joint. In very early cases, in which the only evidence of disease is a slight amount of stiffness and limitation of movement, strapping with Scott's dressing spread on leather, and then the application of a fine bandage over all, is the plan employed. By this means considerable fixation, equable pressure, and some counter-imitation is maintained, and this is kept up until no symptoms remain.

Much more efficient, however, is the application of some much more rigid apparatus, and for this purpose the joint is surrounded with a coating of cotton wool, kept in place by a firmly-applied domett bandage, and then over this enough plaster of Paris or silicate bandage is put on to make a firm, hard case. If it is obtainable, silicate is much to be preferred, as it is much lighter and less bulky. The advantages of using this form of apparatus are that it is cheap, and can be applied in out-patient practice; but to both plaster and silicate there is this strong objection, that they so cover the knee that it cannot be examined without necessitating a fresh splint. Two other forms of apparatus are therefore in constant use; but in order to apply them the patient is usually taken into the hospital so that they may be properly fitted. These are the leather, or poroplastic splint, and Thomas's knee splint.

The leather and poroplastic splints are made in exactly the same way, excepting that the method of softening the material differs. At St. Bartholomew's

LEATHER KNEE SPLINT.

Hospital it generally falls to the duty of the dresser of the case to make this form of splint, though sometimes they are made by an instrument maker.

The way they are made is as follows: A paper pattern is first taken of the shape of splint necessary to encase the leg, from about four inches above the knee to a little above the ankle, a space being cut out to fit the patella. A piece of material is then cut to this shape and softened, the leather by being soaked overnight in dilute acetic acid and water, the poroplastic by being heated in hot water or steam just before use. The material is then firmly applied to the leg with a bandage, so as to make it fit all the hollows and curves of the joint, another bandage having been previously put on the leg to protect the skin. As soon as it is set, the leather is taken off, and any further trimming that may be necessary is done, after which it is lined with wash leather, which is made to adhere by having soap plaster spread on it. In order to increase the rigidity, an iron bar is not infrequently riveted down the back of the splint, and a further advantage is gained by making this bar long enough to fit into a slot in the heel of the boot, thus conveniently maintaining the splint in position. After it is finished so far, it may be applied by having holes punched, into which eyelets are put, so that it can be laced up the front; or, what is perhaps better, webbing straps may be used, as seen in the diagram. This form of splint is most comfortable, and in every way efficient.

The well-known Thomas's knee splint is another form of splint which is much used at St. Bartholomew's. The essential parts of it are, a ring at the top, to surround the limb at the hip, and a smaller ring below to rest on the ground. These are connected by two straight bars of iron. Between these two, a strip of chamois leather is generally stretched. The upper hoop is padded by having flannel bandages twisted, round it to a sufficient thickness, over which chamois leather is stitched. The pad must be made

PATTEN.

THOMAS'S KNEE SPLINT.

especially thick on the inner side, as on this part of the apparatus the tuber ischii rests, through which the body-weight is transmitted to the splint. A webbing strap passes over the opposite shoulder and prevents the splint from slipping off. It is applied by means of bandages which are first fixed to one of the straight bars, and then surround the whole leg and splint, these are applied to the thigh and leg, leaving the knee itself exposed; the patient gets about by a patten on the other foot. One of the great advantages of this splint is that by its means the patient is able to get about, without at the same time bearing any weight on the diseased joint; whereas, by the plaster or leather splints, as a rule, he bears some weight on the diseased limb.

Very frequently when cases are first seen at the hospital there is too much deformity present for the immediate application of a splint of this kind. The leg is in a position of flexion with some external rotation, and displacement of the head of the tibia backwards. No force is, of course, applied in overcoming this malposition, but it is remedied as far as it can be by extension. In using extension, one important principle is observed, this is, that the line of action of the weight must be in the direction of the long axis of the tibia, and in order to accomplish this the leg is elevated on pillows, &c., until the axis of the leg is parallel to the mattress; the pulley of the gallows is adjusted to such a height that the weight pulls in this direction, as seen in the diagram.

The position of the pulley is then from day to day gradually lowered as the deformity gives way. Deformity is also sometimes treated by putting on a Thomas's knee splint, the weight of the leg then acts as extension.

We must now look at what are considered indications for operative interference. These are the formation of abscesses, the presence of sinuses, with much disease of bone, the presence of deformity which cannot be treated by extension, and the gradual constitutional failure of the patient. The various operations practised are incision, arthrectomy, excision, and amputation, about each of which a little must now be said.

*Incision* means merely the opening of abscesses as they form; they are not generally opened particularly early, and when the operation is done, of course all possible precautions are taken to keep the abscess sweet. The patient is kept in bed, with the limb in a splint, until the abscess closes or contracts to a sinus, when he may again be allowed about with an apparatus, such as a leather splint, with a window to permit the dressing of the wound. Sometimes a larger operation is done under the name of incision, viz., making two large openings into the joint, one on each side of the patella, and removing as much unhealthy tissue as can be reached, leaving plenty of opening for drainage, and then fixing the joint up with a splint, with plenty of dressing, which is left on for some weeks, in the hope at the end of that time of finding the wounds healed.

*Arthrectomy* is really modified excision, and consists in attacking all the diseased tissue, and removing it by means of forceps, scissors, and Volckmann's spoons; it is rather a prolonged operation, as it must be done thoroughly to be of any use. We do not think it is as popular an operation as it was at St. Bartholomew's. The class of cases in which it is done are those in which there is deformity, considerable in amount, or when the circumstances of the patient forbid prolonged treatment by rest. It is, of course, most important to obtain perfect healing by first intention, and so antiseptic precautions are rigidly enforced, and cases are not often treated by this method when sinuses are already present. After the operation the limb is put up by one of the methods to be described when speaking of excision.

*Excision* consists in removal of the diseased ends of tibia and femur by sawing, and the rest of the infiltrated tissue as in arthrectomy. At St. Bartholomew's, the view taken of the operation is that this proceeding should not be undertaken till rest has been fairly and fully tried. The steps of the operation need not be described here; but one or two points may be mentioned. At St. Bartholomew's the patella is practically always removed. In finishing the operation, the ends of the bones are generally firmly fixed together, either by two antiseptic ivory pegs, which are driven from the head of the tibia into the femur, through holes drilled ready to receive them, or else by steel pins like sharpened knitting needles. These pegs are removed when they get loose, which generally occurs in about four or five weeks, the ivory ones may, however, be left in permanently. After the operation the limb is put up in one of several ways. A most commonly used splint is a Gant, which consists of a back splint extending from the buttock to the ankle, and an outside splint with a footpiece, having an interruption at the knee and extending to the great trochanter. (See diagram.) Some-

GANT'S SPLINT.

times a further addition is made of a piece which extends up to the axilla, like a long Liston; this is hinged to the rest of the splint at the hip, so that the leg can be slung. A great advantage of the back splint is that by its means the tibia can, by the aid of pads, be more easily levelled up to the femur. Another much-used splint is a shallow MacIntyre, with an interruption at the knee, and a moveable plate behind the joint for it to rest on, which can be taken away for dressing. (See diagram.) By some surgeons the joint is at once put up in plaster of Paris bandages applied over the dressing. Provided the wound be clean, the joint is not dressed for perhaps a week, or even two. When

Movable Knee Piece.

SPLINT FOR EXCISION INTERRUPTED AT THE KNEE.

all is quiet, and some union has taken place between the bones, a leather or a Thomas's knee splint is put on. For a prolonged period after the wound is healed, and the patient getting about, it is necessary for him to wear some apparatus, as even after union has taken place flexion may occur at the junction of the shaft and epihypsis of the femur, until these are united by bone.

*Amputation* is only resorted to when other measures are of no avail; for example, when the bones are too extensively diseased for an excision, or when the patient is sinking from hectic, profuse suppuration, and amyloid disease. Amputation in such cases, obviously, is the only resource to save the patient's life.

2. *Constitutional Treatment.*—Unfortunately patients treated at a hospital like St. Bartholomew's are unable, owing to want of means, to carry out this important part of the treatment, which does not consist so much in drugs as in fresh air and good food. What is often done, however, is to take them into the hospital and fit them with a splint, and then send them for three weeks or a month to the convalescent home at Swanley, in Kent. Sometimes it is found possible to get them to the Margate Infirmary for a longer or shorter period, or they are advised, if they have friends in the country, to go and visit them. As to drugs, Cod liver oil, Syrupus Ferri, Phosphatis Co, Vinum Ferri, and Maltine are the ones usually prescribed.

After any operation patients are, as soon as possible, got out into the fresh air, and in the summer on a fine afternoon many strumous patients may be seen lying out in the square on stretchers and couches. This open-air treatment promotes healing, and greatly benefits the appetite.

## Figures and Tables

**Figure f1:**
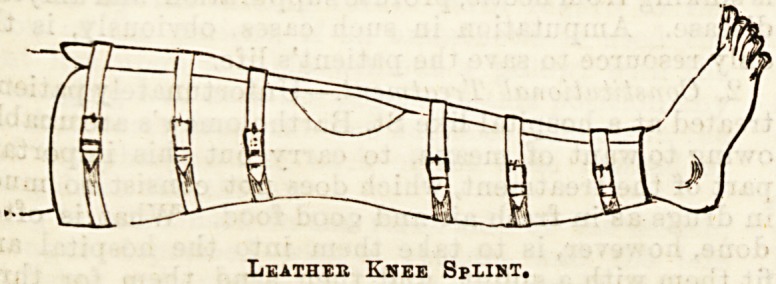


**Figure f2:**
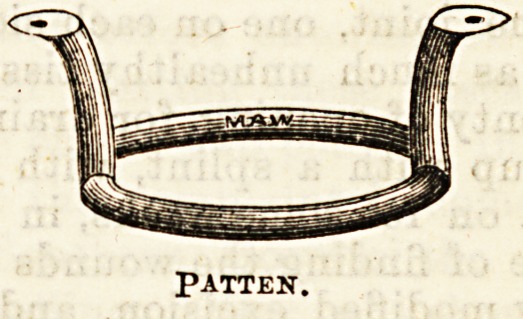


**Figure f3:**
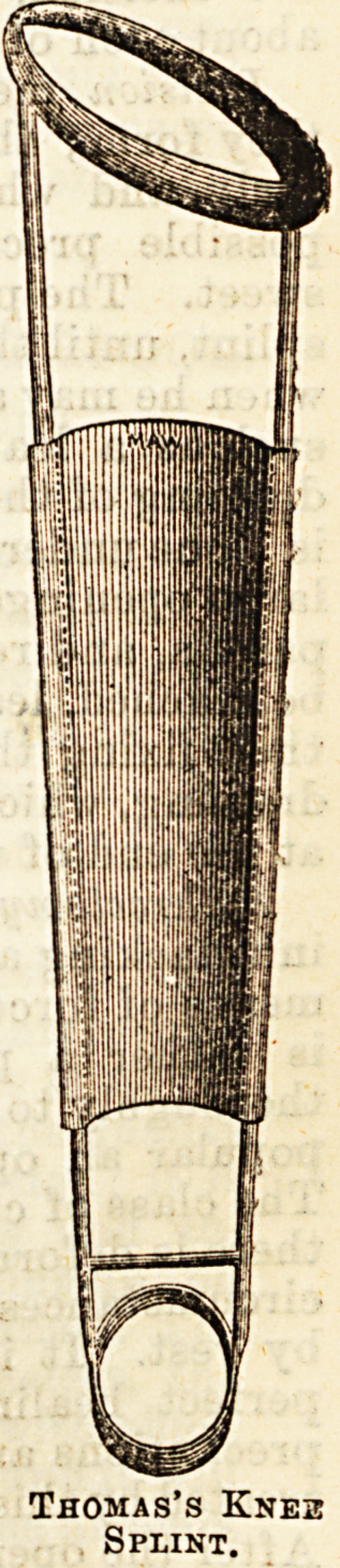


**Figure f4:**
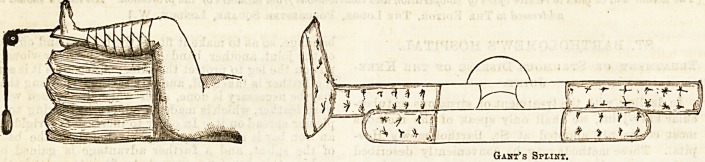


**Figure f5:**